# Examining Pregnant Veterans’ Acceptance and Beliefs Regarding the COVID-19 Vaccine

**DOI:** 10.1007/s11606-022-07588-0

**Published:** 2022-08-30

**Authors:** Kristin M. Mattocks, Aimee Kroll-Desrosiers, Tiffany A. Moore Simas, Lori A. Bastian, Valerie Marteeny, Lorrie Walker, Kate Sheahan, A. Rani Elwy

**Affiliations:** 1 VA Central Western Massachusetts Healthcare System, Birch Building, Room 112, 421 North Main Street, Leeds, MA 01053 USA; 2 University of Massachusetts Medical School, Worcester, MA USA; 3 VA Connecticut Healthcare System, West Haven, CT USA; 4 Yale University School of Medicine, New Haven, CT USA; 5 Durham VA Medical Center, Durham, NC USA; 6 Center for Healthcare Organization and Implementation Research, VA Bedford Healthcare System, Bedford, MA USA; 7 Department of Psychiatry and Human Behavior, Warren Alpert Medical School, Brown University, Providence, MA USA

**Keywords:** Veterans, COVID-19, vaccine, perception, engagement

## Abstract

**Background:**

Pregnant persons have received mixed messages regarding whether or not to receive COVID-19 vaccines as limited data are available regarding vaccine safety for pregnant and lactating persons and breastfeeding infants.

**Objective:**

The aims of this study were to examine pregnant Veteran’s acceptance of COVID-19 vaccines, along with perceptions and beliefs regarding vaccine safety and vaccine conspiracy beliefs.

**Design and Participants:**

We conducted a cross-sectional survey of pregnant Veterans enrolled in VA care who were taking part in an ongoing cohort study at 15 VA medical centers between January and May 2021.

**Main Measures:**

Pregnant Veterans were asked whether they had been offered the COVID-19 vaccine during pregnancy, and whether they chose to accept or refuse it. Additional questions focused on perceptions of COVID-19 vaccine safety and endorsements of vaccine knowledge and conspiracy beliefs. Logistic regression was utilized to examine predictors of acceptance of a vaccine during pregnancy.

**Key Results:**

Overall, 72 pregnant Veterans were offered a COVID-19 vaccine during pregnancy; over two-thirds (69%) opted not to receive a vaccine. Reasons for not receiving a vaccine included potential effects on the baby (64%), side effects for oneself (30%), and immunity from a past COVID-19 infection (12%). Those who received a vaccine had significantly greater vaccine knowledge and less belief in vaccine conspiracy theories. Greater knowledge of vaccines in general (aOR: 1.78; 95% CI: 1.2–2.6) and lower beliefs in vaccine conspiracies (aOR: 0.76; 95% CI: 0.6–0.9) were the strongest predictors of acceptance of a COVID-19 vaccine during pregnancy.

**Conclusions:**

Our study provides important insights regarding pregnant Veterans’ decisions to accept the COVID-19 vaccine, and reasons why they may choose not to accept the vaccine. Given the high endorsement of vaccine conspiracy beliefs, trusted healthcare providers should have ongoing, open discussions about vaccine conspiracy beliefs and provide additional information to dispel these beliefs.

## INTRODUCTION

COVID-19 vaccines to prevent SARS-CoV-2 disease first became available in mid-December 2020, and since that time, more than 165 million people in the USA have received at least one dose of one of the three vaccines available to Americans, representing nearly 62% of the adult population^1^. However, despite the surge in adult vaccinations across the USA, vaccinations among pregnant and lactating persons have lagged, largely due to limited human data on safety during pregnancy that was available at the time of the Food and Drug Administration’s Emergency Use Authorization (EUA)^2^. Recently, two small studies^3,4^ have examined vaccine efficacy in pregnant persons and found that antibodies were produced against SARS-CoV-2, and those antibodies were present in infant cord blood and breast milk, suggesting some COVID-19 protection to infants early in life.

Due to limited available data to weigh the benefits and risks of COVID-19 vaccines during pregnancy, providers must consider patients’ specific risks of SARS-CoV-2 exposure when making vaccine recommendations to pregnant persons. The Centers for Disease Control and Prevention (CDC)^5^ and the Advisory Committee on Immunization Practices (ACIP), in collaboration with the American College of Obstetricians and Gynecologists^6^, the American Academy of Pediatrics, and the Society for Maternal-Fetal Medicine^7^, have recommended that COVID-19 vaccines should not be withheld from pregnant persons^6^. Conversely, early recommendations from the World Health Organization (WHO) suggested that only pregnant women at high risk of exposure to SARS-CoV-2 (e.g., health workers) or those who have comorbidities should consider vaccination^8^.

Given limited COVID-19 vaccine safety information and mixed clinical guidance, pregnant women may experience uncertainty or hesitancy to accept the COVID-19 vaccine. Vaccine hesitancy refers to delay in acceptance or refusal of vaccinations despite the availability of vaccine services^9^. Vaccine acceptance hinges on comprehensive vaccine safety information, and due to the limited data regarding pregnant women, acceptance may be lower in this population. One large multinational study found that 60–70% of pregnant women indicated they would be willing to get vaccinated against COVID-19; education level and employment status were associated with vaccine hesitancy^10^. A similar study in Qatar found a hesitancy rate of 25%, and the main factor determining hesitancy was vaccine safety concerns^11^. A multi-center cross-sectional study in China indicated the vaccine acceptance rate was 77%, with acceptance rates associated with high levels of knowledge regarding COVID-19 and perceived susceptibility to COVID-19^12^. A survey of American persons undergoing obstetric ultrasounds found that 58% would accept COVID-19 vaccine during pregnancy if it were offered, with refusing citing fears of birth defects, vaccine safety, and vaccine side effects^13^.

Previous research focusing on non-pregnant persons suggests that medical mistrust, defined as distrust of health care providers, the health care system, medical treatments, and the government as a steward of public health, prevalent among Black Americans, plays a substantial role in COVID-19 vaccine hesitancy^14,15^. Other studies have suggested that vaccine hesitancy has been associated with authoritarian political views^16^, as well as conspiratorial, religious, or paranoid beliefs^17,18^. Low perceived threat of COVID-19 and political affiliation are also strong predictors of the extent of vaccine hesitancy^13,19^. Individuals living in rural areas, and those with lower household incomes and levels of education have also been found to demonstrate greater COVID-19 vaccine hesitancy^20^.

The purpose of this study was to examine pregnant Veterans’ decisions to accept or decline the COVID-19 vaccine when it was offered by their clinical providers during pregnancy and to understand reasons for refusal including vaccine conspiracy beliefs.

## METHODS

This study was conducted under the auspices of the Center for Maternal and Infant Outcomes Research in Translation (COMFORT) and described in detail previously^21,22^. Pregnant Veterans at 15 VA medical centers were identified by local VA study teams based on their local procedures, for example, when a pregnancy consult was placed in the electronic medical record (EMR) by the Veteran’s primary care or women’s health provider. Recruitment was initiated with mailed invitation packets, including an introductory letter describing the study, and followed by research team telephone calls to potential participants. We conducted telephone surveys (~45 min in length) with women Veterans at 20 weeks of pregnancy and 3 months postpartum. All survey data were collected using Research Electronic Data Capture (REDCap), a secure, web-based application designed to support data capture for research studies^23^. Participants were given US$25 in the form of gift cards for completing each study interview. COMFORT was approved by the Veterans Administration Central Institutional Review Board.

Questions related to COVID-19 vaccine hesitancy were added to our prenatal and postpartum surveys in January 2021 to better understand women’s acceptance of COVID-19 vaccines, perceptions of the safety of COVID-19 vaccines, and their endorsement of vaccine conspiracy beliefs. Overall, 101 women completed the COVID-19 survey between January and May 2021. We asked women if they had “been offered, or had the opportunity to receive, the COVID vaccine during your pregnancy?” with responses including “Yes, I have been offered the vaccine and I accepted the vaccine,” “Yes, I was offered or had the opportunity to receive the vaccine and I decided against it,” and “No, I have not been/was not offered the vaccine during pregnancy.” For this analysis, we only included women who were offered the vaccine and either accepted it (*n*=22) or refused it (*n*=50). We excluded 29 women who were not offered vaccines during pregnancy, though according to US professional societies and the CDC, they should have been offered COVID-19 vaccines during pregnancy^5^–^7^.

The COVID-19 survey asked women about their personal perceptions of risk from COVID-19 (“I am at high risk of becoming seriously ill from COVID-19,” “The coronavirus is a significant threat to my health,” and “I am very concerned that I could die from COVID-19,” measured on a 5-point Likert scale from “strongly agree” to “strongly disagree”); how much they trusted VA primary care providers, obstetricians or midwives, government websites such as the CDC, and internet or social media sites for vaccine advice (“How much do you trust the following sources for vaccine advice?” with responses including “None,” “Some,” or “A lot”); and reasons for not accepting the vaccine (as applicable).

Additionally, we included two scales, the Vaccination Knowledge Scale (VKS)^24^ and the Vaccine Conspiracy Beliefs Scale (VCBS)^25^. The VKS includes 9 items that ask about vaccines in general, with responses of “correct,” “incorrect,” and “do not know.” Items were developed to include only those that had a clear correct or incorrect answer based on available scientific evidence. For this analysis, scores were summed where a “correct” answer was equal to 1 and an “incorrect” or “don’t know” answer was equal to 0. Higher sums indicate greater vaccine knowledge. The VCBS is a 7-item scale that assesses vaccine-specific conspiracy beliefs with participants indicating how much they agree or disagree with a given statement on a 7-point scale that ranges from “strongly disagree” (1) to “strongly agree” (7). For this analysis, scores were summed with responses scored as strongly agree=5, agree=4, unsure=3, disagree=2, and strongly disagree=1. Higher sums indicate greater belief in vaccine conspiracy theories.

Demographics were obtained from both the COVID-19 survey (area of the country where the participant resides [West, Midwest, Northeast, South], education level [high school or less, some college/associate’s degree, 4-year college degree, post-graduate degree], annual household income [<$60,000, $60,000–99,999, $100,000 or more], political party affiliation [Republican, Democrat, Independent, other], and whether they follow news sources for COVID-19 updates) as well as the COMFORT pregnancy survey (age at time of prenatal interview, race [White, Black, and/or Other race], ethnicity [Hispanic or Latinx], marital status [married or single/divorced/widowed], number of children living at home, employment [full/parttime or unemployed/homemaker/student/other], VA service–connected disability [yes or no], current government insurance [yes or no], current private insurance [yes or no], and past self-reported diagnoses of depression, anxiety, and/or posttraumatic stress disorder (PTSD) [yes or no]). We also compared the gestational age and postpartum age (as applicable) between vaccine acceptance/refusal groups, as we thought the amount of time the vaccines were available may have played a role in some pregnant Veterans’ decisions to receive a vaccine or not.

## STATISTICAL ANALYSIS

To compare demographics and other characteristics between Veterans who received and those who refused COVID-19 vaccines, we used chi-squared and Fisher’s exact tests with categorical data and Student’s *t*-tests (with Sattherwaite adjustments when necessary) with continuous data. Next, to examine which of the factors were associated with vaccine hesitancy in our sample, we conducted a complete-case logistic regression predicting the odds of being offered a COVID-19 vaccine and accepting it during pregnancy. We included significant demographic factors (*p*<0.05) from our bivariate analyses, including age, government insurance, education (dichotomized as high school/some college vs. 4-year college or more), and household income (dichotomized as <$60,000 vs. ≥$60,000 per year) as covariates in our models. Then, we ran separate logistic regression models for personal perception of health risk questions (all dichotomized as strongly agree/agree vs. unsure/disagree/strongly disagree), trust in vaccine advice from VA primary care providers and government websites like the CDC (both dichotomized as none vs. some/a lot) and the VKS and VCBS summed scores, adjusted for the demographic covariates. We present adjusted odds ratios (aOR) and 95% confidence intervals (95% CI). All statistical analyses were conducted in SAS version 9.2 (SAS Institute, Inc., Cary, NC).

## RESULTS

Overall, 69% of pregnant Veterans who were offered a vaccine during pregnancy refused. Those who refused were younger (*p*<.0001), less educated (*p*=0.001), and had lower household incomes (*p*=0.04; Table 1). There was no difference in the timing of when the COVID-19 survey was completed between the accepted and refused groups (*p*=0.98; data not shown). Personal perceptions of health risks from COVID-19 also differed between accepted and refused groups; Veterans who strongly agreed or agreed that COVID-19 was a personal health risk were more likely to receive the vaccine. A substantial proportion (64%) of women who refused did so because they worried about the impact of a COVID-19 vaccine on their baby, while 30% worried about the side effects on themselves. Six women indicated a past COVID-19 infection as their reason for not receiving the vaccine. Those who indicated they were unsure or unlikely to receive a COVID-19 vaccine following pregnancy (*n*=28) indicated their fears related to breastfeeding following COVID-19 vaccination (46%), fears related to side effects of vaccines on them (39%), and the belief that they were immune to COVID-19 following a previous infection (21%) (data not shown).

**Table 1:** Demographics by COVID Vaccine Receipt During Pregnancy (*n*=72)

	**Offered and accepted (*n*=22)**		**Offered and refused (*n*=50)**		***p*-value**
**Demographics**	**Mean ± SD**		**Mean ± SD**		
Age at prenatal interview, years	35.9 **±** 2.7		31.5 **±** 5.0		**<.0001**
Pregnant, weeks (*n*=67)	27.2 **±** 6.9		26.4 **±** 6.7		0.67
Postpartum, weeks (*n*=5)	2.5 **±** 2.1		6.0 **±** 3.6		0.32
	***N***	**%**	***N***	**%**	
Race					
White (vs. not white)	13	59.1%	27	54.0%	0.69
Black (vs. not Black)	4	18.2%	18	36.0%	0.13
Other (vs. not other)	7	31.8%	12	24.0%	0.49
Ethnicity: Hispanic or Latinx	4	18.2%	9	18.0%	0.99
Married	15	68.2%	31	62.0%	0.61
Number of children living at home					
0	7	31.8%	17	34.0%	0.92
1	9	40.9%	18	36.0%	
2+	6	27.3%	15	30.0%	
Employment					
Employed full- or part time	16	72.7%	27	54.0%	0.14
Unemployed/homemaker/student/other	6	27.3%	23	46.0%	
Service-connected disability	18	81.8%	38	76.0%	0.76
Government insurance	2	9.1%	18	36.0%	**0.01**
Private insurance	11	50.0%	15	30.0%	0.12
Past diagnoses					
Depression	12	54.6%	39	58.0%	0.78
Anxiety	8	36.4%	24	48.0%	0.36
PTSD	7	31.8%	16	32.0%	0.99
Tested positive for COVID-19 during pregnancy or postpartum	1	4.6%	9	18.0%	0.16
Region of the country					
West	4	18.2%	4	8.0%	0.48
Midwest	3	13.6%	7	14.0%	
Northeast	5	22.7%	10	20.0%	
South	9	40.9%	29	58.0%	
Education					
High school or less	0	0.0%	3	6.0%	**0.001**
Some college/associate’s degree	3	13.6%	21	42.0%	
4-year college degree	7	31.8%	18	36.0%	
Post-graduate degree	12	54.5%	6	12.0%	
Annual household income					
<$60,000	4	18.2%	20	40.0%	**0.04**
$60,000-$99,999	5	22.7%	14	28.0%	
$100,000 or more	12	54.5%	12	24.0%	
*Declined to answer*	*1*	4.5%	*4*	8.0%	
Political party affiliation					
Republican	3	13.6%	9	18.0%	0.11
Democrat	8	36.4%	7	14.0%	
Independent	5	22.7%	9	18.0%	
Something else	0	0.0%	3	6.0%	
No preference	6	27.3%	22	44.0%	
Follows any news source for COVID-19 updates	18	81.8%	31	62.0%	0.10
**Perception of personal health risk**					
I am at high risk of becoming seriously ill from COVID-19.					
Strongly agree/agree	13	59.1%	5	10.0%	**<.0001**
Unsure/disagree/strongly disagree	9	40.9%	44	88.0%	
The coronavirus is a significant threat to my health.					
Strongly agree/agree	15	68.2%	12	24.0%	**0.001**
Unsure/disagree/strongly disagree	7	31.8%	37	74.0%	
I am very concerned that I could die from COVID-19.					
Strongly agree/agree	10	45.5%	5	10.0%	**0.001**
Unsure/disagree/strongly disagree	11	50.0%	43	86.0%	

*Note: “declined to answer” household income was not included in *p*-value calculations. News sources included ABC, CBS, CNN, Fox News, NBC News, NPR, social media sites, other sources

Vaccine hesitancy varied according to trust of information sources (Table 2). Those who refused the vaccine were less likely to trust vaccine information from their primary care doctor or information from government websites such as the CDC. Those who refused a vaccine were also more likely to want to know whether research had been conducted on pregnant women and whether women were more likely to experience a miscarriage or other pregnancy-related problems. Those who received a COVID-19 vaccine had higher scores on the Vaccination Knowledge Scale (7.3 vs. 5.2, *p*=0.001; Table 3) and lower scores on the Vaccine Conspiracy Beliefs Scale (13.0 vs. 18.6, *p*<0.001; Table 4).

**Table 2:** Trust in Sources of Vaccine Information

	**Offered and accepted (*n*=22)**		**Offered and refused (*n*=50)**		
**How much do you trust the following sources for vaccine advice?**	***N***	**%**	***N***	**%**	***p*-value**
Your VA primary care provider					
None	1	4.5%	7	14.0%	**0.03**
Some	3	13.6%	17	34.0%	
A lot	15	68.2%	17	34.0%	
Your OB/midwife					
None	2	9.1%	6	12.0%	0.18
Some	3	13.6%	18	36.0%	
A lot	14	63.6%	23	46.0%	
Government websites like CDC					
None	3	13.6%	11	22.0%	**<.0001**
Some	3	13.6%	23	46.0%	
A lot	16	72.7%	8	16.0%	
Internet and social media sites					
None	8	36.4%	28	56.0%	0.09
Some	10	45.5%	11	22.0%	
A lot	2	9.1%	2	4.0%	
What additional information do you wish you had about the COVID-19 vaccine as it relates to your pregnancy?*					
Whether any research has been conducted with COVID-19 on pregnant women	12	54.5%	41	82.0%	**0.02**
Whether pregnant women in clinical trials experienced any miscarriages or other pregnancy-related problems	11	50.0%	40	80.0%	**0.02**
I would like to know what the Centers for Disease Control (CDC) recommends for pregnant women	10	45.5%	25	50.0%	0.80
Whether the vaccine affects my baby	11	50.0%	37	74.0%	0.06
Whether the vaccine is safe for nursing mothers	10	45.5%	38	76.0%	**0.02**
I don’t need any additional information	7	31.8%	8	16.0%	0.21

*Note: participants were invited to select all that applied for this item

**Table 3:** Vaccination Knowledge Scale

	**Offered and accepted (*n*=22)**		**Offered and refused (*n*=50)**		
**Vaccination Knowledge Scale** **(*N*** **and % correct response)**	***N***	**%**	***N***	**%**	***p*-value**
1. Vaccines are not needed because diseases can be treated (e.g., with antibiotics (-)).	21	95.5%	41	82.0%	0.16
2. Without broadly applied vaccine programs, smallpox would still exist.	21	95.5%	39	78.0%	0.09
3. The effectiveness of vaccines has been proven.	20	90.9%	42	84.0%	0.71
4. Children would be more resistant to diseases if they were not given so many vaccines. (-)	21	95.5%	30	60.0%	**0.002**
5. Diseases like autism, multiple sclerosis, and diabetes might be triggered through vaccinations. (-)	19	86.4%	24	48.0%	**0.004**
6. The immune system of children is overloaded if they are given too many vaccinations. (-)	16	72.7%	21	42.0%	**0.02**
7. If many vaccines are given too early, the child's immune system will not be developed properly. (-)	15	68.2%	21	42.0%	0.05
8. The doses of chemicals used in vaccines are not dangerous for humans.	14	63.6%	19	38.0%	0.05
9. Vaccinations increase the occurrence of allergies. (-)	14	63.6%	23	46.0%	0.10
	**Mean ± SD**		**Mean ± SD**		
Vaccination Knowledge Scale Sum	7.3 **±** 1.9 (2–9)		5.2 **±** 2.6 (0–9)		**0.001**

Note: (-) denotes items with an incorrect statement that were reversed for the scale sum. Scores were summed where a “correct” answer=1 and an “incorrect” or “don’t know” answer=0. Higher sums indicate greater vaccine knowledge

**Table 4:** Vaccine Conspiracy Beliefs Scale

	**Offered and accepted (*n*=22)**		**Offered and refused (*n*=50)**		
**Vaccine Conspiracy Beliefs Scale**	**N**	**%**	**N**	**%**	***p*-value**
1. Vaccine safety data is often fabricated.					
Agree	1	4.5%	9	18.0%	**<.0001**
Unsure	1	4.5%	11	22.0%	
Disagree	9	40.9%	29	58.0%	
Strongly disagree	11	50.0%	1	2.0%	
2. Data about vaccine effectiveness is often fabricated.					
Agree	2	9.1%	8	16.0%	**<.0001**
Unsure	2	9.1%	8	16.0%	
Disagree	8	36.4%	33	66.0%	
Strongly disagree	10	45.5%	1	2.0%	
3. Immunizing children is harmful, and this fact is covered up.					
Agree	0	0.0%	5	10.0%	**0.002**
Unsure	1	4.5%	8	16.0%	
Disagree	8	36.4%	29	58.0%	
Strongly disagree	13	59.1%	8	16.0%	
4. Drug companies cover up the dangers of vaccines.					
Strongly agree/agree	2	9.1%	20	40.0%	**<.0001**
Unsure	2	9.1%	12	24.0%	
Disagree	10	45.5%	17	34.0%	
Strongly disagree	8	36.4%	1	2.0%	
5. People are deceived about the effectiveness of vaccines.					
Strongly agree/agree	6	27.3%	21	42.0%	**0.002**
Unsure	2	9.1%	9	18.0%	
Disagree	6	27.3%	19	38.0%	
Strongly disagree	8	36.4%	1	2.0%	
6. People are deceived about vaccine safety.					
Strongly agree/agree	6	27.3%	19	38.0%	**0.01**
Unsure	1	4.5%	8	16.0%	
Disagree	9	40.9%	22	44.0%	
Strongly disagree	6	27.3%	1	2.0%	
7. The government is trying to cover up the link between vaccines and autism.					
Strongly agree/agree	0	0.0%	6	12.0%	**0.01**
Unsure	2	9.1%	9	18.0%	
Disagree	8	36.4%	26	52.0%	
Strongly disagree	12	54.5%	9	18.0%	
	**Mean ± SD**		**Mean ± SD**		
Vaccine Conspiracy Beliefs Scale Sum	13.0 **±** 5.1 (7–25)		18.6 **±** 4.4 (12–30)		**<.0001**

Note: Scores were summed with responses scored as strongly agree=5, agree=4, unsure=3, disagree=2, strongly disagree=1. Higher sums indicate greater belief in vaccine conspiracy theories

Our logistic regression models indicated that the perceptions that “I am at high risk of becoming seriously ill from COVID-19” (aOR: 9.08; 95% CI: 1.80–45.80), “The coronavirus is a significant threat to my health” (aOR: 9.89; 95% CI: 1.71–57.05), and “I am very concerned that I could die from COVID-19” (aOR: 7.45; 95% CI: 1.22–45.56) all predicted receipt of a COVID-19 vaccine during pregnancy. Similarly, higher scores on the Vaccination Knowledge Scale (aOR: 1.66; 95% CI: 1.07–2.56) and lower scores on the Vaccine Conspiracy Beliefs Scale (aOR: 0.76; 95% CI: 0.62–0.93) significantly predicted receipt of a COVID-19 vaccine during pregnancy. Trust in VA providers or the CDC and government websites for vaccine advice were not significant predictors of whether a participant was likely to receive a vaccine during pregnancy (Table 5).

**Table 5:** Odds of Accepting a COVID-19 Vaccine During Pregnancy

	Adjusted for age, government insurance, education level, and income*		
	**aOR**	**95% LCI**	**95% UCI**
I am at high risk of becoming seriously ill from COVID-19 (agree vs. disagree)	9.08	1.80	45.80
The coronavirus is a significant threat to my health (agree vs. disagree)	6.94	1.57	30.69
I am very concerned that I could die from COVID-19 (agree vs. disagree)	7.45	1.22	45.56
Trust VA Doc for vaccine advice (none vs. some/a lot)	1.16	0.30	4.46
Trust CDC/govt websites for vaccine advice (none vs. some/a lot)	0.33	0.07	1.45
Vaccination Knowledge Scale Sum	1.76	1.17	2.64
Vaccine Conspiracy Beliefs Scale Sum	0.76	0.62	0.93

*Demographic covariates were significant variables from bivariate comparisons between accepted/refused vaccine groups

Note: Each row represents a separate logistic regression model

## DISCUSSION

In our study, 69% of pregnant Veterans receiving community-based obstetrical care were offered, and refused, a COVID-19 vaccine. Though the rate of vaccine refusal in our study is higher than in other recent studies of vaccine hesitancy among pregnant women^13,26^, our study is among the first to examine decision-making among pregnant women who have been offered a vaccine. A majority of pregnant persons in our study cited a lack of scientific information regarding COVID-19 vaccines and the potential for miscarriages, fear of vaccine transmission through breastfeeding, and other infant concerns as reasons for refusing the vaccines. We found no significant differences according to political party affiliation, geographic area of the country, or type of news source (e.g., Fox News, NPR) regarding willingness to accept the COVID-19 vaccination. We also did not find any statistically significant differences in acceptance of the COVID-19 vaccine based on racial/ethnic group.

Our study found that pregnant women who refused a COVID-19 vaccine were more likely to endorse vaccine conspiracy theories than women who accepted the vaccine. Women in our study who refused a COVID-19 vaccine were more likely to believe vaccine data were fabricated, vaccine safety and efficacy data were falsified, and that the government played a role in covering up the linkage between autism and childhood vaccinations. Women who refused vaccinations had little trust in vaccine information provided by their VA primary care providers or the CDC. Women who refused COVID-19 vaccinations also did not believe that COVID-19 posed a significant threat to their health. Taken together, these findings suggest that pregnant women who refused COVID-19 vaccination were generally distrustful of COVID-19 information from pharmaceutical companies, health care providers, and government health agencies.

Our study also reveals concerning findings regarding pregnant Veteran’s perceptions of childhood vaccinations based on their views of COVID-19 vaccinations. Pregnant Veterans who refused a COVID-19 vaccination were more likely to endorse childhood vaccine hesitancy in their beliefs that children would be more resistant to diseases if they were not given so many vaccines, diseases like autism, multiple sclerosis, and diabetes might be triggered through vaccinations, and the immune system of children can be overloaded if they are given too many vaccinations. Previous studies have examined multiple reasons for parental vaccine hesitancy, including lack of sufficient knowledge about vaccines^27^ and negative attitudes towards immunizations^28^, the for-profit nature of pharmaceutical companies^29^, and beliefs that their child is not at risk of serious vaccine-preventable illness^30^. Parents’ hesitancy, refusals, and delays in routine vaccinations are responsible for significant numbers of un-vaccinated or under-vaccinated children, as well as disease outbreaks, comorbidities (meningitis, HPV-related cancers), and untimely deaths^31^. VA primary care providers as well as community obstetricians should take the opportunity to extend conversations of COVID-19 vaccination preferences to childhood vaccination plans with women who are hesitant to accept the COVID-19 vaccine, as this may be an important point of intervention to promote childhood vaccinations or dispel myths regarding the harm of childhood vaccinations.

One promising finding from our study is the degree to which women trust the information provided by their VA primary care provider. Because women remain a numerical minority in the VA, with women representing less than 10% of all enrolled Veterans in VA care, all obstetrical care is paid for by VA but provided by community obstetrical care providers. Previous studies have demonstrated that many women Veterans remain engaged with VA primary and mental health care during their pregnancies^21^, and thus the potential exists for VA primary care providers to play a role in educating pregnant Veterans about COVID-19 vaccine safety during pregnancy.

In VA healthcare, the National Center for Health Promotion and Disease Prevention (NCP), the program office responsible for enterprise-wide COVID-19 vaccination allocation, distribution, and implementation, has developed communication strategies to address COVID-19 concerns related to vaccine acceptance. Drawing on principles of motivational interviewing and patient-centered care, NCP has partnered with VA researchers from the Quality Enhancement Research Initiative (QUERI) to develop a plan for one-on-one conversations with Veterans to learn more about their reasons for vaccine hesitancy. Based on interview and survey data with over 1200 Veterans^32^ collected between January and June 2021, a three-step plan for engaging Veterans in conversations in order to help them move to vaccine acceptance consists of (1) asking Veterans’ permission to have a conversation about what they think about vaccination; do not assume that any VA staff member or provider knows why a Veteran may be hesitant about receiving a vaccine; (2) share with Veterans that other Veterans have said that they received a vaccine to protect their family, friends, other Veterans, their community members, and themselves; and (3) build on the trust that Veterans have with VA healthcare, and involve those providers who Veterans indicate they trust the most in these conversations^33^.

Our study was limited by the relatively small sample size and by survey participation limited to women who had already enrolled in the larger COMFORT study, and therefore may not be generalizable to the larger population of pregnant Veterans or other pregnant persons. Due to the small sample size, we did not see any statistically significant differences by race or ethnicity, though other studies of vaccine hesitancy have found substantial differences in hesitancy by race/ethnicity^15^. Another limitation was that our study was conducted between January and May 2021 when vaccines were first available to the public, and therefore women in our study may have not had enough confidence in the vaccine as they might have if the study were conducted at a later time.

Despite these limitations, this study provides important insights regarding pregnant Veterans’ decisions to accept COVID-19 vaccines, particularly when limited information is available regarding safety during pregnancy and breastfeeding. Future studies should examine vaccine hesitancy once further safety data becomes available and should focus on populations and communities where vaccine acceptance has been lower (e.g., racial/ethnic minorities and inner-city neighborhoods). Finally, the VA should continue to develop strategies to communicate with Veterans regarding COVID-19 vaccines but should work in unison with recommendations from CDC and ACOG when counseling pregnant Veterans on receiving a COVID-19 vaccination.
